# A Narrative Review of Ginkgo Biloba Extract: Biological Function, Molecular Mechanisms, and Applications in Animal Production

**DOI:** 10.3390/antiox15020251

**Published:** 2026-02-14

**Authors:** Mengfan Yao, Lu Liu, Zhihui Hao, Jianzhong Shen, Chongshan Dai

**Affiliations:** 1 Technology Innovation Center for Food Safety Surveillance and Detection (Hainan), Sanya Institute of China Agricultural University, Sanya 572025, China; 2 State Key Laboratory of Veterinary Public Health and Safety, Department of Veterinary Pharmacology and Toxicology, College of Veterinary Medicine, China Agricultural University, Beijing 100193, China

**Keywords:** Ginkgo biloba extract, feed additives, animal production, molecular mechanisms

## Abstract

Ginkgo biloba extract (GBE), obtained from dried Ginkgo biloba leaves, provides a natural option. GBE supplementation can increase livestock’s productivity through various biological functions, such as combating oxidative stress, reducing inflammation, optimizing gut microbiota, detoxifying intestinal toxins, and regulating immune responses. In this review, we utilized keywords such as “Ginkgo biloba extract” or “Ginkgo biloba extract” and “animal production” or “animal nutrition” to gather research on its various biological functions and the underlying mechanisms from databases such as Web of Science and PubMed, up to December 2025. Then, we systematically summarize the main bioactive components of GBE, its beneficial effects in livestock at different life stages and during different production cycles, and the related molecular pathways. Additionally, safety assessments and the potential applications were also discussed. This review highlights that GBE may be an effective plant-derived feed additive with multiple functions and strong potential to improve animal health, production efficiency, and product quality under intensive farming conditions. We hope that this review can stimulate broader discussions and better develop and utilize GBE as a feed additive in animal production.

## 1. Introduction

The use of feed additives in animal production has long been integral to enhancing growth performance, disease prevention, and feed efficiency. Historically, antibiotics served as the cornerstone of such additives due to their potent antimicrobial and growth-promoting effects. However, decades of indiscriminate antibiotic use have precipitated severe global crises, including the proliferation of antimicrobial resistance (AMR), which threatens both veterinary and human medicine [1,2]. Residual antibiotics in animal products further raise food safety concerns, while their non-targeted action disrupts gut microbiota, impairing intestinal immunity and nutrient absorption [3]. Consequently, regulatory bodies worldwide, exemplified by the European Union’s 2006 ban on antibiotic growth promoters, have imposed stringent restrictions, necessitating sustainable alternatives [4]. In this context, herbal feed additives derived from medicinal plants have emerged as scientifically validated, eco-compatible solutions [5]. Unlike synthetic antibiotics, herbal feed additives exert multi-targeted bioactivities, including antimicrobial, anti-inflammatory, antioxidant, and immunomodulatory effects through synergistic interactions among phytochemicals (e.g., polyphenols, alkaloids, and essential oils) [5]. Their residues degrade naturally, minimizing environmental persistence and mitigating food chain contamination. Critically, herbal feed additives can maintain gut health homeostasis by modulating microbial diversity and enhancing epithelial barrier function without promoting AMR [6]. Moreover, their adaptability to organic farming systems aligns with the global shift toward “green” animal husbandry [7].

Ginkgo biloba extract (GBE) is a standardized product obtained through scientific extraction and enrichment processes from Ginkgo biloba leaves [8]. Its primary bioactive constituents include flavonoid compounds (such as quercetin, kaempferol, isorhamnetin, and their glycosides) and terpenoid lactones (such as ginkgolides A, B, C, and bilobalide) [9]. Modern pharmacological research has extensively confirmed that these compounds exhibit multifaceted biological functions, including remarkable antioxidant activity, anti-inflammatory effects, anti-microbial activity, microcirculation improvement, and neuroprotection [10,11,12]. These core functionalities not only establish the significant role of GBE in human medicine, particularly in preventing and treating cardiovascular/cerebrovascular diseases and neurodegenerative disorders, but also provide a theoretical foundation for its application in enhancing animal health and production performance.

**Figure 1:**
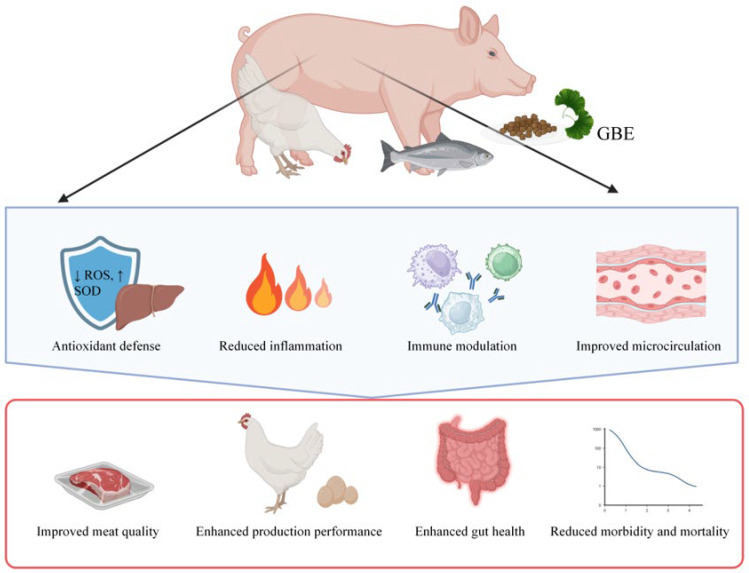
Schematic mechanism of the effects of GBE on physiological functions and production performance in livestock. GBE is proposed to enhance antioxidant defense, attenuate inflammatory responses, modulate immune function, and improve microcirculation. Collectively, these physiological improvements contribute to enhanced production performance and meat quality, optimized gut health, and reduced morbidity and mortality.

Recently, some studies have shown that GBE supplementation can effectively improve livestock production via multifaceted biological activities, including anti-oxidative stress, anti-inflammatory effects, improvement of gut microbiota, neutralization of intestinal toxins, and immune regulation [13,14,15]. For instance, Wu et al. showed that GBE treatment at 75 μg/mL exhibited strong activity against the biofilm formation of Salmonella and Listeria isolates from poultry [15]. Yang et al. showed that 0.12% GBE supplementation in the diet can effectively alleviate fatty liver hemorrhagic syndrome in laying hens via reshaping gut microbiota [13]. Mechanically, studies have indicated that GBE may exert beneficial effects through (1) mitigating oxidative stress damage to protect tissues and organs (especially liver, intestines, and reproductive system); (2) suppressing inflammatory responses, alleviating subclinical inflammatory states, improving intestinal barrier function, and maintaining gut microecological balance; (3) regulating immune function by enhancing innate and adaptive immunity to increase disease resistance; (4) improving blood circulation to facilitate nutrient transport and metabolic waste clearance, thereby indirectly boosting tissue vitality and production performance; (5) eliminating harmful pathogens and neutralizing harmful environmental toxins. These potential effects are expected to translate into tangible production benefits, including enhanced feed conversion ratio (FCR), accelerated growth, improved reproductive performance (e.g., semen quality, follicular development), increased stress resistance, and reduced morbidity and mortality rates (Figure 1).

In this review, we utilized keywords such as “Ginkgo biloba extract” or “Ginkgo biloba extract” and “animal production” or “animal nutrition” to gather research on its various biological functions and the underlying mechanisms from databases such as Web of Science and PubMed, up to December 2025. Then, we systematically summarize the main bioactive components of GBE and its application effects and research progress across different physiological stages and production phases in livestock (such as swine, poultry, and ruminants). We also summarize its safety and optimal utilization strategies. We hope this review can help us deepen scientific understanding of the roles of GBE in livestock nutrition and health, develop efficient, safe, and eco-friendly novel functional feed additives, and advance the livestock industry toward greater efficiency, health, and sustainability.

## 2. The Main Active Components of GBE

GBE is from the dried leaves of Ginkgo biloba L., using suitable chemical solvents, and it enriches various active components [16]. As of now, over 200 compounds have been identified in GBE. The detailed information has been addressed in a previous review by Liu et al. [17]. Notably, flavonoids and lactones make up about 40% of the extract and are regarded as its primary active ingredients. Moreover, GBE also contains a variety of substances, such as organic acids, proanthocyanidins, polyisoprene alcohols, polysaccharides, and proteins [17].

### 2.1. Flavonoid Compounds

**Figure 2:**
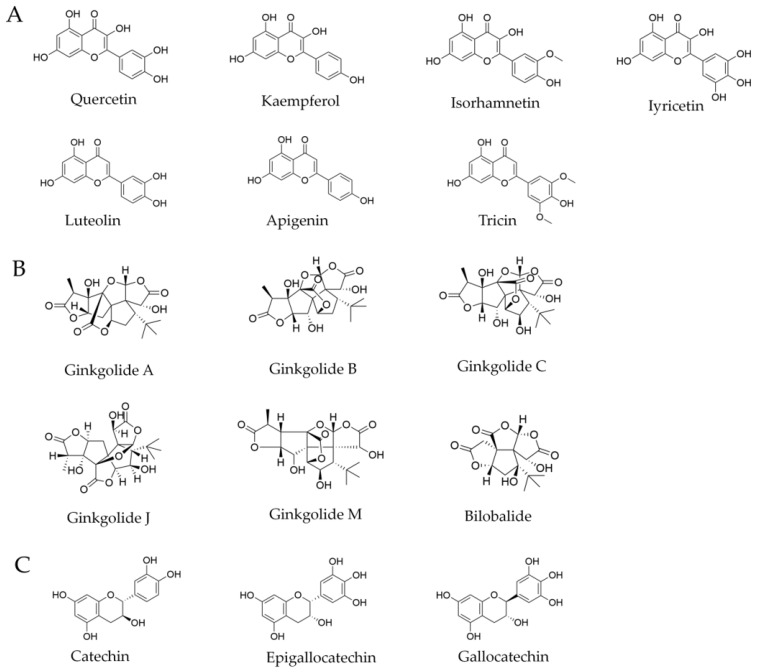
The structures of the main active components in GBE. (**A**) Flavonoid compounds, including quercetin, kaempferol, isorhamnetin, iyricetin, luteolim, apigenim, and tricin. (**B**) Ginkgolide compounds (Ginkgolide A, B, C, M, J) and Bilobalide are all terpenoid natural products with a cage-like structure. (**C**) Proanthocyanidins, including catechin, epigallocatechin, and gallocatechin, belong to flavanol polyphenols.

Currently, nearly 40 flavonoid components have been identified in GBE. They are mainly categorized into flavones (also known as aglycones), flavanols, catechins, and other groups, accounting for approximately 30% of the active ingredients in GBE [18]. Among them, flavones can be further classified into seven types based on differences in their aglycone structures, including quercetin, kaempferol, isorhamnetin, myricetin, luteolin, apigenin, and tricin. Notably, quercetin, kaempferol, and isorhamnetin are the main aglycone scaffolds in the flavanol glycosides of GBE (Figure 2A) [19]. Despite subtle differences in their chemical structures, these variations determine the strength and specificity of their biological activities.

According to international standards (such as EGb 761), the flavonoid content must be at least 24%, with quercetin, kaempferol, and isorhamnetin listed as key indicators for testing. The flavonoid structure contains a 5,7,4-trihydroxy group and a 3-hydroxy group connected to a sugar moiety [20]. Through these phenolic hydroxyl groups, flavonoids neutralize free radicals, endowing them with potent antioxidant properties and effectively protecting cell membranes and DNA from oxidative damage.

Bioflavonoids are another distinctive class of chemical components in GBE. It was formed by the connection of two flavonoid monomers (typically apigenin or its derivatives) through carbon–carbon or carbon–oxygen–carbon bonds [21]. These compounds have a relatively narrow distribution across the plant kingdom, and their unique spatial conformation confers a pharmacological activity profile distinct from that of monomeric flavonoids [22]. The bioflavonoids in GBE mainly include six types: 5-methoxy-deginkgolide, 7-deginkgolide, ginkgoflavone, isoginkgoflavone, ginsenoside bioflavonoid, and cephalotaxus bioflavonoid [23]. Their core characteristic is the presence of a unique dimeric flavonoid backbone. Additionally, flavanol glycosides can significantly enhance their water solubility through glycosylation modifications, which is of great importance for improving their bioavailability [24]. In addition to the two core components mentioned above, GBE also contains other flavonoid-related substances, including catechins, proanthocyanidins, and dihydroflavonols (e.g., dihydromyricetin). Although they are not used as standardized quality control indicators, their presence undoubtedly plays a synergistic role in enhancing the overall biological activity of the extract, as they are effective antioxidants.

### 2.2. Terpenoid Lactone Compounds

Terpenoid compounds refer to natural compounds with the general formula (C5H8)n, along with their oxygen-containing and differently saturated derivatives. These compounds are primarily derived from isoprene or isopentane through various linkages and are mainly divided into two subgroups: ginkgolides (i.e., diterpenoid compounds) and bilobalide (i.e., a sesquiterpenoid compound) [25]. Ginkgolides are a type of diterpenoid trilactone compound with a 20-carbon atom backbone [26]. Their molecular structure is highly complex and rigid, consisting of six five-membered rings (including three lactone rings, one spiro[4.4]nonane carbon ring, and one tetrahydrofuran ring), with a unique tert-butyl group. This highly functionalized cage-like structure allows them to bind with specific biological targets with high affinity [27]. The main ginkgolides that have been isolated and identified include ginkgolides A, B, C, J, M, and bilobalide (Figure 2B), which are all diterpenoid lactone compounds [28]. They share the same core backbone, with differences only in the number and position of hydroxyl groups. These subtle structural variations significantly affect their biological activity strength [29]. Bilobalide is another important terpenoid lactone in Ginkgo biloba leaves. For example, although bilobalide’s antagonism of platelet-activating factor (PAF) is not as potent as ginkgolide B, Bilobalide exhibits unique neuroprotective effects [25].

### 2.3. Proanthocyanidin Compounds

Proanthocyanidins (PACs), also known as condensed tannins, are a class of polyphenolic compounds formed by the polymerization of flavan-3-ol units [30]. They are colorless in their natural state but can undergo cleavage under acidic heating conditions to produce colored anthocyanidins. Phytochemical studies on GBE have confirmed that the primary monomeric units constituting its PACs are flavan-3-ols, including catechin, epigallocatechin, and gallocatechin (Figure 2C) [31]. The structural differences in these monomers, primarily the number of hydroxyl groups on the B-ring, determine the type and properties of the final polymer. Additionally, multiple studies on the reference extract EGb 761 consistently show that its proanthocyanidin content stabilizes around 7% [30]. This level of content makes the PACs component quantitatively comparable to the entire terpenoid trilactone component (approximately 6%), highlighting its significant role in the overall mass balance of the extract.

### 2.4. Other Compound Classes

GBE incorporates various alkylphenolic acids, notably ginkgolic acid (GA) and ginkgo phenol. GA stands out as the predominant component, representing roughly 80% of the total alkylphenolic acid content. This compound, a derivative of salicylic acid, is chiefly sourced from Ginkgo biloba leaves, fruits, and outer seed coats. Key constituents feature GA, hydrogenated GA, hydrogenated ginkgolic subacid, and ginkgolic new acid [32]. These elements demonstrate potent antimicrobial, antiparasitic, HIV protease inhibitory, and potential anticancer activities [33,34,35]; yet, they introduce hazards like sensitization, cytotoxicity, and mutagenicity [36]. As a result, international pharmacopeias enforce rigorous caps on GA concentrations in extracts; for example, the Chinese Pharmacopoeia restricts it to ≤7.0 μg/g. Purification methods are thus vital to lower its levels and safeguard the extract’s safety.

Polyisoprenol compounds in GBE consist of extended chains built from isoprenyl units and terminal isoprenol groups, with most occurring as acetate esters [37]. These polyisoprenols manifest broad pharmacological effects, including effectively countering liver damage triggered by carbon tetrachloride and alcohol, reducing transaminase levels, boosting cell membrane fluidity and permeability, and showcasing antitumor, hematopoietic-promoting, and antioxidant actions.

Additionally, GBE also contains a diverse array of organic acids, including fatty acids, hydroxy acids, amino acids, glucaric acid, shikimic acid, and 6-hydroxy kynurenic acid (6-HKA). Among these, 6-HKA acts as a wide-ranging antagonist against central nervous system neurotransmitters such as N-methyl-D-aspartate (NMDA) and α-amino-3-hydroxy-5-methyl-4-isoxazolepropionic acid (AMPA), thereby reducing symptoms of brain hypoxia. Studies have also shown that GBE contains various amino acids. High-performance liquid chromatography (HPLC) analysis revealed that the total amino acid content in dried Ginkgo biloba leaves is as high as 92.26 mg/g, with eight essential amino acids accounting for 46.9% of the total amino acids [38]. In addition, GBE has also been reported to contain compounds such as 4′-O-methylpyridoxine and polysaccharides [39], with specific functions yet to be further studied.

## 3. The Bioavailability and Metabolism of GBE

The bioavailability and metabolism of the active components in GBE are regulated by multiple factors within the animal body. Both human and animal studies consistently show that ginkgolides and bilobalide are well absorbed after oral administration. Their absolute bioavailability is quite high, with ginkgolide A around 80%, ginkgolide B around 88%, and bilobalide around 79%, and food intake does not affect their bioavailability [40]. After intravenous injection, the elimination follows a biphasic pattern, with an initial half-life of 0.3 h (peak time 10 min) and a terminal half-life of 1.5 h, with primary excretion through urine (40–50%) [41]. Flavonoid glycosides (such as quercetin and kaempferol) require enzymatic hydrolysis by gut microbiota to be converted into aglycones. The microbiota further breaks down the aglycones’ cyclic structure, converting them into a series of smaller, more easily absorbed phenolic acid metabolites, such as phenylacetic acid and 3-hydroxyphenyl propionic acid [42]. Their bioavailability is significantly influenced by microbial metabolic activity. For example, antibiotic intervention can inhibit microbial metabolism, thereby increasing the bioavailability of certain flavonoids like isosorbide but decreasing the conversion efficiency of other flavonoid glycosides [43]. The biological activity of ingested Ginkgo flavonoids largely depends on the “secondary processing” by the individual’s gut microbiota. Due to significant differences in gut microbiota composition and metabolic capacity between individuals, this means that even when the same dose of GBE is consumed, the active metabolite profiles and concentrations in different individuals may differ significantly [42]. The extraction process of GBE directly determines the release of active components. Methanol extracts have the strongest antioxidant activity due to their high flavonoid/phenolic content, while n-hexane extracts enrich hydrophobic components such as ginkgoid acid, showing strong antimicrobial activity but with higher cellular toxicity risks [44].

The bioavailability of the active components in GBE is regulated by multiple factors, including extraction methods, formulation techniques, microbial metabolism, and drug transport proteins. Terpenoid lactones, such as ginkgolide A and B, have relatively high oral bioavailability, with their metabolism primarily excreted through urine [45]. Flavonoid glycosides, on the other hand, must be metabolized by the gut microbiota into aglycones before they can be absorbed, and their bioavailability is significantly affected by microbial metabolic activity. Additionally, new formulation technologies such as phospholipid complexes can enhance the bioavailability of terpenoid lactones, further optimizing their pharmacokinetic properties [43].

## 4. The Biological Activities of GBE and Molecular Mechanisms

### 4.1. The Inhibition of Oxidative Stress, Mitochondrial Dysfunction, and Apoptosis

In livestock and aquaculture, oxidative stress is a common constraint under heat stress, crowding, pathogen pressure, and dietary contaminants. These stressors can impair growth, FCR, and tissue integrity. During biological metabolism, free radicals are continuously generated as reactive species characterized by strong oxidative properties, enabling them to react readily with biological macromolecules such as proteins, lipids, and nucleic acids. Under normal physiological conditions, organisms regulate these species through enzymatic antioxidants (such as superoxide dismutase [SOD], glutathione peroxidase [GPX], and catalase [CAT]) and non-enzymatic systems to maintain dynamic redox homeostasis [46,47]. When this balance is disrupted by environmental insults, nutritional imbalance, or age-related declines in antioxidant capacity, oxidative stress ensues. Excessive free radicals lead to lipid peroxidation, protein dysfunction, and DNA damage, ultimately driving cell apoptosis and tissue injury [48].

Studies have shown that flavonoids are the primary constituents in GBE responsible for free radical scavenging and antioxidant activities. The free radical scavenging capacity of flavonoids mainly depends on their reductive phenolic hydroxyl groups. Using the DPPH assay, Fadi et al. reported an IC_50_ of ~15.5 μg/mL for GBE, and fractionation identified multiple polyphenolic constituents with stronger radical-scavenging capacities than BHT (IC_50_ = 17.3 μg/mL) [31] Ahmad et al. systematically compared the antioxidant activities of GBE obtained using different solvents. The results showed that the methanol extract exhibited the strongest activity in the DPPH radical scavenging assay, achieving an inhibition rate of up to 82.8%, which was significantly higher than that of the ethanol extract (76.7%) and extracts from other plants tested in parallel (e.g., Stevia rebaudiana) [49]. Zhang et al. isolated and compared the activities of four representative functional components from GBE, namely ginkgo flavonoids (GF), ginkgolides (G), oligomeric proanthocyanidins (OPC), and organic acids (OA). The study reached a clear conclusion that, in terms of DPPH radical scavenging capacity, the activity ranking was OPC > GF > OA > G. In contrast, for ABTS cation radical scavenging, the activity ranking was GF > OPC > OA > G [50]. This refined analysis clearly demonstrates that polyphenolic compounds, such as flavonoids and proanthocyanidins, are the primary contributors to the direct free radical scavenging activity of GBE.

Studies have confirmed that GBE can effectively scavenge superoxide anion radicals (O_2_^−^), hydroxyl radicals (•OH), and peroxyl radicals (ROO•) [51]. Ellnain-Wojtaszek et al. (2003) clearly indicated that this scavenging capacity is mainly attributable to the high flavonoid content of GBE [52]. Maitra et al. conducted a landmark study on the standardized extract EGb 761. Using multiple in vitro models, EGb 761 is a highly efficient scavenger of peroxyl radicals [53]. With advances in computational chemistry, Zeppilli et al. demonstrated through quantum chemical calculations that ginkgolides and bilobalide, which are unique to Ginkgo biloba, are potent scavengers of peroxyl and alkoxyl radicals. Their mechanism involves hydrogen-atom transfer (HAT), with thermodynamic and kinetic parameters comparable to those of the classical antioxidant trolox, a water-soluble analog of vitamin E [54]. This discovery clearly established the important role of terpenoid lactones in direct antioxidant activity, overturning the traditional view that they act mainly through other pharmacological pathways, and substantially enriching our understanding of the antioxidant mechanisms of GBE. In addition to directly reacting with free radicals, GBE exerts antioxidant effects through the enhancement of the endogenous enzymatic antioxidant defense system. Multiple studies report that GBE or standardized EGb 761 can increase activities of antioxidant enzymes (SOD/CAT/GPX) and reduce lipid peroxidation in stressed models [55,56,57]. For example, Bridi et al. found that the supplementation of EGb 761 via oral administration at a dose of 100 mg/kg body weight for 14 consecutive days significantly increased the activities of SOD and catalase (CAT) in the hippocampus, striatum, and substantia nigra of brains in a rat neurodegenerative disease model [55].

Nrf2, being the key transcription factor for antioxidant responses in stressed animal cells, then moves to the nucleus. In the nucleus, it forms heterodimers with small Maf proteins. These heterodimers bind to the antioxidant response elements (ARE) in the promoter regions, initiating the transcription of various antioxidant genes. These genes include GST, SOD, CAT NAD(P)H quinone dehydrogenase 1 (NQO1), and heme oxygenase-1 (HO-1) [58]. This coordinated gene expression establishes a multi-level defense system for cells. Liu et al. found that GBE treatment can decrease Keap1 protein and increase the expression of nuclear Nrf2 in liver cells, indicating the activation of the Keap1/Nrf2/ARE signaling pathway [59]. In tilapia, GBE supplementation at the doses of 0.5–4 g per kg feed significantly increased the expression of Nrf2, GST, NQO1, and HO-1 mRNAs in liver tissues [60]. Grypioti et al. reported that treatment with ginkgolide B (BN52021) at 10 mg/kg body weight significantly alleviated acute liver injury induced by acetaminophen via the inhibition of oxidative stress, inflammation, and apoptosis in the liver tissues of rats [61]. Additionally, Zheng et al. found that feeding tilapia with 0.5, 1, and 4 g/kg of GBE for 60 days significantly upregulated Nrf2 in liver tissue, which, in turn, activated downstream targets like GST, NQO1, and HO-1. This led to the increases in the activities of T-AOC, SOD, and CAT, and the levels of GSH in serum and liver, effectively reducing glyphosate-induced damage in tilapia [62].

The signaling cascade involving phosphoinositide 3-kinase (PI3K) and protein kinase B (Akt) stands as a pivotal intracellular mechanism for cell survival. Functioning as a central regulator, this pathway harmonizes inputs from growth factors, cytokines, and hormones to govern vital cellular functions, including survival, proliferation, metabolism, and angiogenesis, while robustly inhibiting apoptotic mechanisms. Extensive research has established GBE as a potent activator of the PI3K/Akt pathway [63]. For instance, in rodent models of myocardial ischemia–reperfusion injury, Chen et al. revealed that administration of 20 and 40 mg/kg body weight of EGb 761 can significantly increase the expression of in the heart tissues, following to reduce cardiac damage, suggesting that the activation of the Akt pathway underlies GBE’s cardioprotective benefits against reperfusion damage [64]. Additionally, Akt activation acts as a critical precursor for Nrf2 induction. Akt facilitates Nrf2 stabilization and activation through the phosphorylation and suppression of glycogen synthase kinase-3β, a kinase that promotes Nrf2 breakdown [58]. Consistently, Guo et al. demonstrated in a cerebral ischemia–reperfusion injury model that combined treatment with Ginkgo flavonoids and ginkgolides at a ratio of 4:1 effectively activated the PI3K/Akt/Nrf2 signaling pathway, thereby improving neurological functional deficits [65]. In neuronal cells, a specific PI3K inhibitor, LY294002, can completely block the ginkgolide-induced increase in p-Akt levels, then markedly reduce the upregulation of phosphorylated Nrf2 [65]. Collectively, these insights confirm that GBE’s bioactive elements can initiate PI3K/Akt activation, which then propagates to modulate Nrf2 dynamics.

The anti-apoptotic effects of GBE are further linked to the protection of mitochondrial integrity. The mitochondrial membrane potential (ΔΨm) is a key indicator of mitochondrial function; oxidative stress can disrupt the inner mitochondrial membrane, causing ΔΨm dissipation, a hallmark early event of apoptosis. EGb 761 has been reported to protect against H_2_O_2_-induced ΔΨm loss in cellular systems [66]. The collapse of ΔΨm and the occurrence of mitochondrial outer membrane permeabilization (MOMP) ultimately lead to the release of the key pro-apoptotic factor cytochrome c from the mitochondrial intermembrane space into the cytosol [67]. Cytosolic cytochrome c associates with apoptotic protease-activating factor-1 and pro-caspase-9, forming a multiprotein complex known as the apoptosome in the presence of dATP, which serves as the initiating platform for downstream caspase cascade activation [68]. Shen et al. clearly showed that EGb 761 pretreatment significantly inhibited cytochrome c release from mitochondria in cardiomyocytes subjected to hypoxia–reoxygenation injury [69]. By blocking this critical step, GBE interrupts apoptotic signal amplification at its source. Whether MOMP occurs largely depends on the dynamic balance between anti-apoptotic members (e.g., Bcl-2, Bcl-xL) and pro-apoptotic members (e.g., Bax and Bak) of the Bcl-2 protein family. In a myocardial injury model, Chen et al. found that EGb 761 treatment significantly downregulated the expression of the pro-apoptotic protein Bax while markedly upregulating the anti-apoptotic protein Bcl-2, finally reducing the levels of activated caspase-3, DNA fragmentation, and cell apoptosis in cardiomyocytes [64]. Ye et al. reported that GBE supplementation can effectively inhibit cell apoptosis by increasing nitric oxide (NO) and cyclic guanosine monophosphate (cGMP) levels, activating cGMP-dependent protein kinase (PKG), and suppressing caspase activity [70].

**Figure 3:**
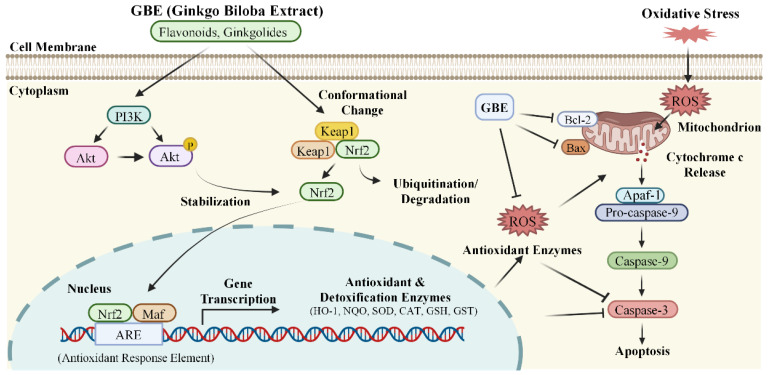
Anti-apoptotic mechanisms of GBE under oxidative stress. GBE, mainly through flavonoids and ginkgolides, activates cytoprotective signaling pathways. GBE stimulates the PI3K/Akt pathway and stabilizes Nrf2 by inhibiting Keap1-mediated degradation. Nuclear Nrf2 induces the expression of antioxidant and detoxification enzymes, including HO-1, NQO1, SOD, CAT, GSH, and GST, thereby reducing intracellular ROS levels. At the mitochondrial level, GBE increases the Bcl-2/Bax ratio and prevents cytochrome C release, which suppresses caspases-3,9 activation. Together, these actions inhibit apoptosis and promote cell survival under oxidative stress. Created with BioRender.com.

In summary, GBE supplementation offers protective effects against oxidative stress, mitochondrial dysfunction, and apoptosis by (i) directly scavenging radicals, (ii) enhancing the body’s endogenous antioxidant defenses, and (iii) activating the PI3K/Akt and Nrf2/ARE pathways (Figure 3). These effects are primarily attributed to key active ingredients such as flavonoids and ginkgolides. Additionally, activation of the PKG pathway partially contributes to GBE’s regulation of oxidative stress and cell apoptosis, though the detailed molecular mechanism still needs further investigation. From a livestock production perspective, engaging Nrf2-dependent antioxidant defenses can be a strategy to increase resilience against common stressors in modern farming, such as heat stress, weaning, high stocking density, and contaminants, thus improving feed efficiency and growth. However, practical use requires strict standardization of extracts, accurate dose determination, and careful evaluation of safety margins specific to each species.

### 4.2. The Inhibition of Inflammatory Responses

In production systems, inflammation often reduces performance. It can appear during enteric challenges, vaccination, mycotoxin exposure, and heat stress. This section discusses how GBE may help control inflammation. Kotakadi et al. reported that standardized EGb 761 reduced intestinal inflammation in a mouse colitis model. In vitro, treatment with 100 μg/mL of EGb 761 was shown to inhibit the expression of iNOS, eNOS, and COX-2 in ANA-1 cells in a time-dependent manner [71]. In a rat myocardial ischemia–reperfusion model, Chen et al. revealed that EGb 761 supplementation at 20 and 40 mg/kg body weight markedly activated the Akt/Nrf2 signaling pathway, then upregulated the activities of SOD and GSH-Px, finally suppressing the release of inflammatory mediators such as TNF-α, IL-6, and IL-1β in the heart tissues in a rat myocardial ischemia–reperfusion model [64]. Zhou et al. demonstrated that a polysaccharide fraction purified from GBE exerts anti-inflammatory effects by inhibiting the Toll-like receptor 4 (TLR4)/NF-κB signaling pathway [72]. Ilieva et al. reported that treatment with GBE at 1–100 μg/mL markedly reduced ocular inflammation in endotoxin-related models, as evidenced by lower levels of nitric oxide (NO) and prostaglandin E2 (PGE2) [73]. GBE supplementation at 100 mg/kg body weight for 14 days can reduce epilepsy-induced memory impairment by inhibiting lncRNA-COX2/NF-κB in mouse hippocampal tissues [11]. In a hypoxic hypothyroid mouse model, Adebayo et al. found that GBE treatment at 20 mg/kg body weight for 14 days markedly attenuated oxidative imbalance, which was directly associated with reduced inflammatory mediators and improved vascular function, highlighting its therapeutic potential [74]. An in vitro study showed that treatment with 50 μg/mL of GBE for 18 h scavenges reactive oxygen species, thereby inhibiting NF-κB activation and suppressing the downstream expression of vascular cell adhesion molecule-1 (VCAM-1) and intercellular adhesion molecule-1 (ICAM-1) [75].

Terpenoid lactones in GBE also contribute to anti-inflammatory effects. Many studies highlight platelet-activating factor (PAF) signaling. PAF is a lipid mediator that can amplify inflammation. When GBE blocks PAF signaling, inflammatory cascades can weaken. This may also improve microcirculation, which can indirectly reduce inflammatory burden. Several studies support a specific role of ginkgolides. Han et al. suggested that the terpenoid fraction had stronger anti-inflammatory effects than the flavonoid fraction in an arthritis-related model [76]. Clinical and ex vivo studies also report vascular and platelet-related benefits, which are consistent with PAF inhibition [77,78]. In liver injury models, ginkgolide B showed protective effects that aligned with reduced oxidative stress and inflammation [61].

In summary, studies suggest that GBE can reduce inflammatory responses. The most common signals involve the inhibitory effects of TLR4/NF-κB activity and reduced PAF-related signaling. Flavonoids (e.g., quercetin) and terpenoid lactones (e.g., ginkgolide B) likely contribute together (Table 1). Some studies also link anti-inflammation to PI3K/Akt–Nrf2 activation through improved antioxidant defense. In the future, the precise molecular mechanism and activity-based material basis still need further exploration.

### 4.3. Immunomodulatory Effects

Immune competence is a key production trait. It affects survival, vaccine response, and resilience under husbandry stress in livestock. Cellular immunity is mediated by multiple immune cell types, including T lymphocytes, macrophages, and NK cells, and plays a central role in host defense against infection, tumor surveillance, and immune monitoring. GBE can regulate the functions of these immune cells through multi-target and multi-pathway mechanisms. In Table 2, a summary of immunomodulatory effects of GBE on cellular and humoral immunity was shown.

Xia et al. reported that patients with arsenic poisoning caused by long-term arsenic exposure exhibited immune dysregulation, characterized by an increased proportion of pro-inflammatory Th17 cells and a reduced proportion of anti-inflammatory regulatory T-cells [85]. The study further revealed the underlying molecular mechanism: GBE downregulated mRNA expression of the Th17-specific transcription factor RORγt while upregulating mRNA expression of the Treg-specific transcription factor Foxp3, thereby reshaping T-cell differentiation at the transcriptional level [85]. Hui et al. showed that GBE promotes differentiation of CD4^+^ T cells into Treg cells by inhibiting the hypoxia-inducible factor-1α (HIF-1α)/hexokinase-2 (HK2) signaling pathway, which not only alleviates post-ischemic cerebral inflammation but also reveals a novel immunomodulatory mechanism underlying the neuroprotective effects of GBE [86]. Kotakadi et al. found that the standardized EGb 761 significantly reduced the number of pro-inflammatory effector T cells (CD4^+^/CD25^−^/Foxp3^−^) in colonic tissue. The mechanism involves direct induction of apoptosis in these activated CD4^+^ effector T cells, thereby effectively eliminating “destructive” cells at inflammatory sites and controlling inflammatory responses [71]. In a double-blind, placebo-controlled trial in patients with schizophrenia, Zhang et al. showed that, compared with haloperidol alone, combined treatment with GBE significantly increased the reduced CD4/CD8 T-cell ratio and the number of IL-2-secreting cells, indicating restoration of impaired T-cell immune function [87]. Tian et al. found that ginkgolide B restores Th1/Th2 balance by inhibiting the TLR4/NF-κB pathway, thereby alleviating airway inflammation and hyperresponsiveness and effectively treating house-dust-mite (HDM)-induced allergic asthma in mice [88]. In peripheral blood leukocytes from patients with Alzheimer’s disease, Sochocka et al. observed that GBE treatment reduced the production of both IFN-γ and IL-10, suggesting that GBE may remodel cytokine networks through a complex regulatory mechanism in elderly individuals and patients with neurodegenerative diseases [89]. In addition, GBE enhances NK cell activity. A study by Matsushima et al. in healthy individuals showed that continuous intake of GBE for 14 days significantly increased the cytotoxic activity of peripheral blood NK cells. Flow cytometric analysis revealed increased expression of the NK-cell-specific surface marker CD56 and decreased expression of the B-cell marker CD19, indicating that GBE selectively promotes activation or expansion of the NK-cell population. In vitro experiments further confirmed that GBE at concentrations of 400–800 μg/mL exerts maximal NK-cell-activating effects [90].

B lymphocytes and functions primarily mediate humoral immunity through the production of antibodies to eliminate extracellular pathogens and toxins. GBE also exerts significant regulatory effects on humoral immune function, particularly under conditions of immunosuppression or when enhanced antibody responses are required. In a cyclophosphamide-induced immunosuppressed mouse model, polysaccharide components of GBE exhibited pronounced immunodecorated activity. Jin et al. demonstrated that Ginkgo biloba leaf polysaccharides significantly enhanced the proliferative response of splenic lymphocytes to the B-cell-specific mitogen lipopolysaccharide (LPS) [91]. Xu et al. further reported that polysaccharides isolated from the Ginkgo biloba sarcodes markedly increased serum levels of IgM, IgG, and IgA in immunosuppressed mice, thereby restoring humoral immune function [92]. Melenkova et al. pioneered the evaluation of GBE as a potential mucosal adjuvant. In experiments using bovine serum albumin (BSA) as an antigen to immunize chickens and rabbits, GBE used as an adjuvant significantly enhanced humoral immune responses compared with non-adjuvanted controls, as evidenced by markedly elevated serum IgG and IgM titers against BSA. Importantly, when applied as a mucosal adjuvant, GBE effectively induced the production of secretory IgA in saliva, which is critical for preventing pathogen invasion via mucosal surfaces. In the chicken model, GBE also promoted the accumulation of IgY in egg yolk [93]. Matsushima et al. observed that in healthy individuals, 14 days of GBE supplementation resulted in a reduction in the expression of the B-cell surface marker CD19. Under physiological conditions, 40 mg/mL of GBE may maintain immune homeostasis by mildly downregulating B-cell populations to balance other immune compartments, such as NK cells, whose activity was enhanced in the same study [90]. Regulation of humoral immunity by GBE is also mediated through indirect mechanisms. For example, ginkgolides act as platelet-activating factor (PAF) receptor antagonists [94,95], and since PAF participates in B-cell activation, GBE may modulate B-cell function via PAF antagonism. Likewise, the ability of GBE to induce dendritic cell maturation provides a prerequisite for robust B-cell responses and antibody class switching [96]. Jin et al. further indicated that GBE supplementation at 400 mg/kg body weight via the oral administration for 28 days can restored spleen, thymus, and lymphocyte proliferation indices, secretion and mRNA expression of IL-4, IFN-γ, IL-2, and IL-10, protein expression of TLR2, TLR4, MyD88, and IRAK4, and phosphorylation levels of JNK, ERK, and p38, finally improved cyclophosphamide-induced immunosuppression in mice [91].

In summary, GBE can regulate cellular immunity and humoral immunity through multi-target and multi-pathway mechanisms. In terms of cellular immunity, GBE can reshape T cell differentiation (such as regulating Th17/Treg balance, restoring CD4/CD8 ratio, and Th1/Th2 balance), enhance NK cell activity, and regulate the inflammatory cytokine network. In terms of humoral immunity, GBE and its polysaccharide components can enhance the antibody levels (IgM, IgG, and IgA) of immunosuppressive models, enhance the response of B cells to mitogens, and act as mucosal adjuvants to promote the production of secretory IgA. Its regulatory effects also involve indirect mechanisms such as antagonizing PAF receptors, promoting dendritic cell maturation, and regulating TLR/MAPK signaling pathways. Overall, GBE has a bidirectional regulatory function on the immune system, exhibiting immune regulatory potential under various pathological conditions such as infection, tumors, autoimmune diseases, and neuroinflammation. Importantly, modulation of innate and adaptive immunity provides the most direct nutritional relevance under high disease pressure or environmental stress in production systems.

**Table 2:** Summary of immunomodulatory effects of GBE on cellular and humoral immunity.

Type	Component	Model	Key Findings	Reference
Cellular immunity	Th17/Treg cells	Arsenic patients	Decreases Th17 and RORγt; increases Treg and Foxp3	Xia et al. [85]
	Treg cells	Mouse ischemic stroke	Promotes Treg differentiation (via HIF-1α/HK2)	Hui et al. [86]
	Effector T cells	Mouse colitis	Reduces CD4^+^ effector T cells; induces apoptosis	Kotakadi et al. [71]
	Th1/Th2 cells	Mouse asthma	Inhibits Th2 responses; restores Th1/Th2 balance	Tian et al. [88]
	T-cell subsets	Schizophrenia	Increases CD4/CD8 ratio and IL-2-secreting cells	Zhang et al. [87]
	Macrophages	LPS-activated	Decreases NO, PGE_2_, iNOS, COX-2; inhibits NF-κB	Mir et al. [97]
	Macrophages	Diabetic rat	Restoration of impaired phagocytic activity	Izgüt et al. [98]
	Macrophages	COPD model	Activates autophagy; decreases IL-6 levels	Zhang et al. [99]
	NK cells	Healthy human	Increases cytotoxic activity and CD56 expression	Matsushima et al. [90]
	Dendritic cells	Mouse BMDDCs	Promotes DC maturation and costimulatory markers	Chen et al. [96]
Humoral immunity	B lymphocytes	Immunosuppression	Increases LPS-driven B-cell proliferation	Jin et al. [91]
	B lymphocytes	Healthy human	Decreases CD19 expression	Matsushima et al. [90]
	Serum immunoglobulins	Immunosuppression	Increases serum IgM, IgG, and IgA levels	Xu et al. [92]
	Antibody response	Immunized models	Increases serum IgG/IgM and salivary IgA	Melenkova et al. [93]

### 4.4. Antibacterial, Anti-Virus, and Anti-Parasitic Activities

Multiple studies have demonstrated that GBE exhibits a broad range of antibacterial, anti-viral, and anti-parasitic activities (as shown in Table 3).

GBE and its constituents display pronounced inhibitory effects against various viruses, particularly enveloped viruses. These antiviral effects primarily target the early stages of the viral life cycle, including viral attachment and entry, as well as subsequent replication processes. Haruyama et al. reported that GBE pretreatment at 5 μg/mL marked reduce the infection of influenza A viruses (H1N1 and H3N2 subtypes) and influenza B virus in MDCK cells. The underlying mechanism involves direct interaction of GBE with viral hemagglutinin (HA), thereby disrupting HA–host receptor binding and preventing viral attachment [100]. Wang et al. demonstrated that a nanoemulsion formulated with polyphenols extracted from Ginkgo biloba leaves exhibited up to 70% direct virucidal activity and a 74.9% cellular protection rate against H3N2 [101]. In addition, the flavonoid ginkgetin from Ginkgo biloba has also been reported to possess anti-influenza virus activity [102]. Borenstein et al. identified that GA is a key anti-herpesvirus constituent of GBE. GA suppresses herpes simplex virus type 1 (HSV-1) replication through multiple mechanisms, including inhibition of virion entry by blocking the initial fusion event and suppression of viral protein synthesis after cell entry, with a treatment concentration of 10–50 μM [103]. Bhutta et al. confirmed the in vivo anti-HSV-1 activity of GA using a mouse skin infection model. Topical application of 10 mM GA twice daily for 14 days significantly reduced mortality and disease severity. Notably, GA remained effective against acyclovir-resistant (ACV-resistant) HSV-1, significantly reducing skin lesions where standard acyclovir treatment failed [104]. Hayashi et al. showed that ginkgetin potently inhibited replication of both HSV-1 and HSV-2 by specifically suppressing transcription of viral immediate-early genes [105]. A review by Akanchise et al. indicated that GBE components interfere with the coronavirus life cycle. Ginkgolide A irreversibly inhibits the papain-like protease at 1.79 μM, while quercetin suppresses viral replication by targeting the 3-chymotrypsin-like protease and papain-like protease with a docking energy of 4.62–6.25 kcal/mol [106]. Experimental evidence from Bhutta et al. found that GA exhibits potent inhibitory activity against human coronavirus 229E (HCoV-229E). The study indicated that GA can effectively inhibit the virus at concentrations of 10 μM and 15 μM [107]. Borenstein et al. further revealed the remarkable broad-spectrum antiviral capacity of GAs, demonstrating that 10 μM of GA effectively inhibits the fusion of all three classes of fusion proteins, including HIV, Ebola, Zika, and influenza A, while also inhibiting human cytomegalovirus with an IC_50_ of approximately 6.8–7.3 μM [103]. In a murine model of viral myocarditis, Wang et al. found that GBE treatment at 125 mg/kg administered daily for 30 days alleviated myocardial injury induced by coxsackievirus B3 infection. This protective effect was achieved by reducing viral titers and suppressing inflammatory mediators and fibrosis markers, specifically S100A4 and MMP-3 [108]. In addition, Ginkgo biloba polyprenols exhibited strong in vitro inhibitory activity against hepatitis B virus (HBV) [101]. Clinical studies further suggest that EGb 761 improves hepatic fibrosis and microcirculatory disturbances in patients with chronic hepatitis B virus infection [109]. Notably, Yiu et al. indicated that GBE may induce cytochrome P450 enzymes, thereby accelerating metabolism of the antiretroviral drug efavirenz and potentially leading to therapeutic failure in HIV patients, highlighting the risk of drug–herb interactions [110].

GBE exhibits inhibitory activity against a broad spectrum of Gram-positive and Gram-negative bacteria. Its antibacterial effects are mediated through multi-target mechanisms, including direct disruption of bacterial structures, interference with quorum-related behaviors, and inhibition of key metabolic processes. Ražná et al. reported that Staphylococcus aureus is among the most sensitive bacterial species to ethanol extracts of GBE, with minimum inhibitory concentration values of MIC_50_ = 64.2 µg/mL and MIC_90_ = 72.2 µg/mL [111]. It was also reported that Ginkgo biloba exocarp extract at concentrations of 4–12 μg/mL dose-dependently inhibited biofilm formation by *Staphylococcus aureus* and methicillin-resistant *S. aureus* (MRSA) [112]. He et al. found that GA exhibited an MIC of 4 µg/mL against Streptococcus mutans. At sub-inhibitory concentrations, it effectively suppressed acid production, adhesion to saliva-coated hydroxyapatite, and biofilm formation [113]. Sati et al. reported that methanolic extracts of GBE showed strong antibacterial activity against Bacillus subtilis, producing an inhibition zone of 20 mm and an MIC value of 7.81 µg/mL [114]. Lee et al. demonstrated that 5 μg/mL of GAs (C15:1 and C17:1) and 100 μg/mL of GBE inhibited biofilm formation by pathogenic *Escherichia coli* O157:H7 by suppressing expression of genes involved in curli fimbriae synthesis, without affecting bacterial growth at the tested concentrations [12]. Zhang et al. investigated the antibacterial mechanism of GBE against spoilage bacteria in aquatic products and found that GBE disrupted the integrity of the cell membrane and cell wall of Shewanella putrefaciens, resulting in leakage of intracellular contents (e.g., alkaline phosphatase), increased electrical conductivity, and ultimately bacterial death [115]. Wu et al. showed that GBE at 75 µg/mL significantly inhibited biofilm formation by Salmonella strains isolated from poultry meat, partly by suppressing bacterial swarming motility [15]. Carraturo et al. discovered that phenolic acids and bilobol from Ginkgo biloba’s sarcotesta have strong bactericidal effects against *Salmonella typhimurium*, with MIC and MBC values of 8.3 µg/mL [116].

GBE and its constituents also exhibit inhibitory activity against a range of medically important protozoa and helminths, providing valuable leads for the development of novel antiparasitic agents. Zhang et al. conducted an innovative study combining GBE with the first-line antimalarial drug artemisinin to treat mice infected with Plasmodium yoelii, a widely used animal model of human malaria. The combination regimen was markedly more effective than artemisinin monotherapy, significantly reducing parasitemia, improving malaria-associated microcirculatory dysfunction, and effectively modulating host immune responses. Mechanistic analyses revealed that the combined therapy downregulated the expression of key parasite genes required for erythrocyte invasion, such as apical membrane antigen 1 and merozoite surface protein 1, thereby disrupting the parasite replication cycle [117]. An in vitro study by Ugwu et al. demonstrated that GA (15:1) at concentrations of 0.78–3.125 μg/mL has potent inhibitory activity against *Cryptosporidium andersoni* [118]. Multiple review articles have highlighted the broad-spectrum antiparasitic or antiprotozoal activities of GBE and its constituents. Sati et al. reported that Ginkgo biloba flavonoids possess antiprotozoal activity [16]. Ražná et al. emphasized the well-recognized antiparasitic properties of GBE and their terpenoid constituents [111]. Collectively, these findings provide a strong rationale for considering GBE as a promising source of antiparasitic drug candidates.

**Table 3:** Summary of antimicrobial activities of Ginkgo biloba leaf extract.

Type	Pathogen	Key Effects	Reference
Viruses	Influenza A/B viruses	Inhibition of viral adsorption via interference with HA function	Haruyama et al. [100]
	HSV-1/2	Inhibition of viral fusion, viral protein synthesis, and immediate-early gene transcription	Borenstein et al. [103]
	HCoV-229E	Reduction of viral yield and protein expression; inhibition of viral fusion	Bhutta et al. [107]
	HIV	Inhibition of virus–host membrane fusion	Borenstein et al. [103]
	CVB3	Attenuation of viral myocarditis via suppression of S100A4 and MMP-3	Wang et al. [108]
Bacteria	MRSA	Inhibition of biofilm formation, disruption of mature biofilms, interference with cell wall–associated processes	Wang et al. [112]
	Streptococcus mutans	Inhibition of bacterial growth, acid production, adhesion, and biofilm formation	He et al. [113]
	Bacillus subtilis	Inhibition of bacterial growth	Sati et al. [114]
	Escherichia coli O157:H7	Suppression of biofilm formation via inhibition of curli fimbriae gene expression	Lee et al. [12]
	Salmonella enterica	Bactericidal and bacteriolytic effects; inhibition of biofilm formation	Carraturo et al. [116]
Parasites	*Plasmodium yoelii*	Reduction of parasitemia and suppression of erythrocyte invasion genes (in combination with artemisinin)	Zhang et al. [117]
	*Cryptosporidium andersoni*	In vitro inhibition of parasite growth	Ugwu et al. [118]

## 5. Applications of GBE in Animal Production

### 5.1. Enhancement of Animal Growth Performance

Modern intensive animal production systems, characterized by high stocking density, rapid growth rates, and frequent regrouping, inevitably expose animals to prolonged metabolic and environmental stress. Such persistent stress disrupts the balance between free radical generation and antioxidant defenses, thereby inducing oxidative stress. Oxidative stress represents a common underlying factor limiting growth potential, impairing immune function, and reducing product quality in livestock. The growth-promoting effects of GBE are closely associated with its antioxidant properties, which enhance metabolic efficiency and maintain intestinal function, ultimately translating into improved production performance (Table 4). Numerous studies have confirmed that GBE and its fermented products improve animal health and production performance. In broiler chickens, Niu et al. reported that fermented Ginkgo biloba leaves (FGBL) at 3.5–4.5 g/kg of diet for 42 days increased average daily gain (ADG) and average daily feed intake (ADFI) [119]. The study also reported improved plasma antioxidant capacity (PAC). In a subsequent study, Niu et al. confirmed that supplementation with 3.5 g/kg of diet FGBL for 42 days similarly increased body weight gain and feed intake in broilers [120]. El-Kasrawy et al. found that supplementing drinking water with 0.25 cm/L Ginkgo biloba leaf oil for three consecutive days per week increased body weight, weight gain, and feed intake in broilers, linked to higher insulin-like growth factor-I expression [121]. Additionally, Yan reported that continuous feeding of a diet containing 0.8% GBE for 35 days improved feed conversion efficiency and body weight gain in broilers aged 21–35 days [122]. In swine production, Zhou et al. included fermented Ginkgo biloba leaf residue at 10% in diets for weaned piglets [123]. The treatment increased final body weight, average daily gain, and feed efficiency. The authors reported stronger effects than those in antibiotic-supplemented controls. Zhao et al. found that feeding a diet containing 0.5% of diet FGBL for 8 weeks increased egg-laying rate and improved the feed-to-egg ratio in laying hens [124]. Moreover, studies also confirmed that GBE improves growth performance and immune function in weaned piglets, with enhanced immunity indirectly promoting growth by reducing disease burden [122]. However, during the finishing phase of pigs, the growth-promoting effects appear to be less pronounced. Several studies have reported that supplementation with herbal extracts has limited effects on average daily gain in finishing pigs, although it may contribute to reduced backfat thickness. This suggests that the efficacy of 0.8% GBE in the feed may depend on the animal’s growth stage [125]. In ruminants, Chen et al. studied Haimen White goats during a 70-day fattening period. The authors replaced alfalfa pellets with 18% of the diet fermented Ginkgo biloba leaf residue (GBLR). This replacement increased final body weight, average daily gain, and feed intake. It also reduced the feed-to-gain ratio. Specifically, the average daily gain in the 18% replacement group reached 170.11 g/day, substantially higher than the 140.89 g/day observed in the control group [126]. In aquaculture, Abdel-Latif et al. demonstrated that supplementation of a basal diet with 7.5% GBE for 8 weeks enhanced growth performance and feed utilization efficiency in Nile tilapia (*Oreochromis niloticus*) [60]. Liao et al. found that GBE-supplemented diets alleviated hepatopancreatic damage and improved survival in shrimp. Feeding a diet containing 2 g/kg GBE for 21 days significantly increased total hemocyte count (THC), hemocyanin levels, expression of antioxidant-related genes, and the activities of their corresponding enzymes in Pacific white shrimp (*Litopenaeus vannamei*) [127]. In addition, GBE contains organic compounds such as alcohols, lipids, and ketones. Some of these compounds have volatile, oil-like aromas. These aromas may stimulate digestive secretions and increase appetite. This may increase feed intake and support growth performance.

In summary, evidence from multiple production models suggests that GBE and related products can improve growth performance in several species. Reported outcomes include higher body weight gain, better feed efficiency, and improved production traits such as egg-laying performance. Benefits appear more consistent in broilers, weaned piglets, goats, tilapia, and shrimp, while effects in finishing pigs may be weaker. Many studies link these outcomes to improved antioxidant status and better gut or hepatopancreatic health, and some reports also suggest higher immune competence. In practice, the magnitude of response likely depends on the animal species, growth stage, product form, and inclusion level.

### 5.2. Regulation of Lipid Metabolism and Improvement of Meat Quality

Dysregulation of lipid metabolism can lead to excessive lipid accumulation in animals, which not only impairs normal growth and development and deteriorates meat quality in fattening livestock but also predisposes animals to metabolic disorders such as ketosis (Table 5). Abdel-Zaher et al. reported that oral EGb761 at 100 mg/kg body weight via the oral administration for 28 days reduced serum lipid peroxides and cholesterol in rats. These results suggest anti–lipid peroxidation and hypocholesterolemic effects [128]. It was also found that the dietary supplementation with fermented FGBL or GBE significantly reduced abdominal fat deposition and intermuscular fat width in broilers [122,129]. These effects are closely associated with the regulation of circulating lipid profiles. Ren et al. showed that 0.8 g/kg of diet GBE supplementation for 10 days significantly decreased serum triglyceride (TG) and total cholesterol (TCHO) levels in broilers [130]. In a heat-stressed broiler model, Zhou et al. further demonstrated that supplementation with 600 mg/kg of diet GBE for 21 days reduced lipogenesis by suppressing the AMPK/SREBP-1c/ACC signaling pathway [14]. Niu et al. provided direct evidence in broilers fed 3.5–4.5 g/kg of diet FGBL. The birds showed lower breast muscle lightness values, which indicates a darker color. The birds also showed lower cooking loss in thigh muscle and lower drip loss in both breast and thigh muscles. Importantly, these improvements were accompanied by significantly increased total SOD activity and overall antioxidant capacity, together with a marked reduction in MDA levels in muscle tissues [119]. These findings clearly indicate that GBE establishes a robust antioxidant defense system in vivo, thereby effectively delaying postmortem oxidative deterioration of meat. Other studies have similarly confirmed that dietary inclusion of 0.2–0.7% GBE improves meat pH, reduces drip loss and cooking loss, and ultimately enhances overall meat quality and shelf life [129]. GBE further optimizes fatty acid profiles in animal products by modulating lipid metabolism, thereby aligning these products more closely with human nutritional requirements. Cao et al. reported that broilers fed 0.2–0.7% of diet GBE had lower saturated fatty acids (SFA), such as C16:0 and C18:0, in muscle [129]. The birds also had higher unsaturated fatty acids. The increase was most evident for polyunsaturated fatty acids (PUFA), such as C18:2, C18:3, and C20:4. Similar effects were observed in laying hens. Zhao et al. (2013) reported that supplementation with 0.5% of diet FGBL increased yolk oleic acid (C18:1 n-9), linoleic acid (C18:2 n-6), total PUFA content, and the PUFA/SFA ratio [124]. Comparable results have been reported in pigs and sheep, where herbal feed additives increased PUFA and monounsaturated fatty acid (MUFA) contents while reducing SFA levels in meat [131]. Such optimization of fatty acid composition not only enhances sensory attributes of animal products but also increases their health appeal to consumers.

In summary, evidence indicates that GBE can influence lipid handling in animals, with downstream effects that are commonly evaluated in production research (Table 5). In practice, lipid metabolism outcomes are discussed together with carcass fat deposition, meat quality, and fatty acid profiles. These links provide a clear production-relevant frame for interpreting the reported changes in blood lipids, tissue oxidative stability, and product composition.

### 5.3. Enhancement of Disease Resistance and Stress Tolerance

GBE exhibits pronounced immunomodulatory activity, enabling a comprehensive enhancement of host immune competence and thereby improving resistance to disease and tolerance to environmental and physiological stressors. For example, Yan et al. reported that dietary supplementation with 0.8% or 1.0% GBE increased the bursa of Fabricius index and thymus index in broilers [122]. In birds, the bursa of Fabricius is a key immune organ. It supports B-lymphocyte development and maturation. Proper development of this organ is important for humoral immune responses. Consistently, it also reported that GBE supplementation markedly elevated antibody titers against Newcastle disease virus and increased serum globulin and IgG levels [122]. Together, these findings suggest that GBE supports humoral immunity in broilers. This may help animals develop better protection after vaccination or pathogen exposure.

Heat stress represents a major cause of economic losses in summer livestock production, and GBE exhibits unique protective effects against heat-induced injury, particularly within the cardiovascular system. In poultry, circulatory failure contributes to heat-stress-related mortality. Zhou et al. provided direct evidence that combined supplementation with 600 mg/kg GBE and 300 mg/kg tea polyphenols in the diet effectively mitigates heat-stress-induced impairments in growth performance and metabolic homeostasis in broilers [14]. Zhang et al. further investigated the cardioprotective effects of the specialized formulation EGb 761 under acute heat stress (38 °C for 3 h) in chickens. Their results showed that dietary supplementation with 0.1–0.6% of diet EGb 761 for 45 days significantly alleviated heat-stress-induced clinical abnormalities and myocardial pathological damage. This unique protective mechanism was closely associated with heat shock protein 70 (Hsp70); notably, EGb 761 did not directly induce Hsp70 in cardiomyocytes but selectively upregulated its expression in cardiac microvascular endothelial cells. The induced Hsp70 was subsequently released into the circulation, conferring protection to the cardiac microvasculature against heat-stress-induced injury [132]. Therefore, cardiovascular protection is an important outcome when evaluating GBE under heat stress.

Weaning is a major physiological and psychological stressor for piglets. Zhou et al. reported that 10% of Ginkgo biloba leaf residue in diets helped alleviate weaning stress in piglets, supporting its potential as a functional feed additive [123]. GBE supplementation at 30 mg/kg of diet for 28 days can also increase serum IgG, IgA, and IgM levels while improving the distribution of T-lymphocyte subsets in weaned piglets [133]. The authors linked these effects to better antioxidant defenses. GBE may reduce excessive ROS during stress. This may help protect immune cells and other tissues and support systemic homeostasis.

GBE may modulate immunity in a balanced way rather than acting as a simple stimulant. In Nile tilapia, GBE treatment increased non-specific immune parameters such as complement C3, lysozyme, and IgM, while upregulating the gene expression of key cytokines, including pro-inflammatory IL-1β and TNF-α, as well as anti-inflammatory IL-10 [60]. This bidirectional regulation of pro- and anti-inflammatory cytokines indicates that GBE functions not as a simple immune stimulant or suppressant, but rather as a sophisticated immunological homeostasis modulator. Beyond immune regulation, GBE also exhibits additional biological activities, including hypolipidemic, antithrombotic, anti-fatigue, antitumor, and hypoxia-tolerant effects [134,135,136]. These patterns suggest that GBE supplementation may influence both pro- and anti-inflammatory signaling. This supports the idea that GBE may help maintain immune homeostasis.

In summary, GBE is linked to improved immune competence and better tolerance to common husbandry stressors in several animal models. More standardized comparisons are needed to define optimal inclusion levels and to confirm performance benefits under farm-like conditions.

### 5.4. Maintenance of Intestinal Health and Enhancement of Production Performance

GBE supplementation selectively suppresses the proliferation of pathogenic microorganisms while promoting the colonization of beneficial bacteria, thereby modulating gut microbial composition and maintaining intestinal microecological balance [137]. Wang et al. reported that quercetin supplementation at 0.2 g/kg diet for 42 days changed the cecal microbiota in broilers, i.e., it decreased pathogen-associated genera such as *Salmonella* and *E. coli*, while increasing beneficial genera such as *Lactobacillus* and *Bifidobacterium* [137]. Yang et al. reported that in laying hens with fatty liver hemorrhagic syndrome, GBE supplementation markedly increased *Megasphaera* levels in the cecum and elevated short-chain fatty acids. These changes were linked to better barrier integrity, energy metabolism, and immune homeostasis in the intestines [13]. In addition to modulating microbial composition, multiple studies have demonstrated that GBE markedly improves intestinal morphological characteristics. El-Kasrawy et al. found that supplementation with Ginkgo biloba oil in drinking water increased intestinal villus length in broilers, thereby expanding the absorptive surface area [121]. Prihambodo et al. further demonstrated that dietary supplementation with flavonoids, key active components of GBE, significantly increased villus height and the villus height-to-crypt depth ratio (V/C ratio) in the duodenum and jejunum [138]. These structural improvements facilitate more efficient digestion and nutrient absorption, providing a morphological basis for sustained enhancement of animal growth performance. Niu et al. observed that dietary supplementation with 3.5 g/kg of diet FGBL increased the relative weight of the duodenum in broilers, indirectly indicating enhanced intestinal development and functional capacity [120]. In addition, GBE enhances mucosal defense through bidirectional modulation of immune responses, thereby regulating intestinal immunity and anti-inflammatory processes. Sun et al. provided critical molecular evidence showing that LPS challenge markedly downregulated the expression of tight junction-related genes, including zonula occludens-1 and occludin, in the jejunum of broilers, thereby increasing the risk of intestinal permeability. Dietary supplementation with quercetin effectively reversed these adverse changes, preserving the integrity of the intestinal physical barrier [139]. Moreover, Dong et al. demonstrated that 800 mg/kg of diet quercetin for 11 days alleviated the downregulation of mucin-2 gene expression, thereby maintaining mucus layer thickness and quality and reinforcing the intestinal chemical barrier [140].

In summary, GBE is consistently linked to improving gut status in animal production systems. This link is production-relevant because gut integrity and nutrient use often shape feed efficiency and stress resilience. Current evidence supports a coordinated pattern across microbiota, gut structure, and barrier function, but the strength of the response may vary with species, diet background, and the form and dose of GBE.

### 5.5. Future Development of Feed-Grade GBE: Standardization, Dosing, and Safety Assurance

GBE is increasingly discussed as a functional ingredient for feed, and many studies report benefits on performance and stress resilience. For feed application, practical translation depends on a clear extract definition, realistic inclusion levels, and species-appropriate safety evaluation over the intended feeding period.

In feed industry applications, the standardization of ginkgo-based feed products faces multiple technical and economic challenges. Clearly distinguishing standardized purified extracts (e.g., pharmaceutical-grade EGb 761) from crude preparations is essential; this distinction underpins bioactivity stability and directly affects animal performance and feed safety. Pharmaceutical-grade EGb 761 is governed by stringent specifications (24% flavonoids, 6% terpene lactones, and GAs < 5 mg/kg feed) [141] This standardization supports high reproducibility of effects, yet feed-grade products often deviate due to cost control. Compared with purified extracts, the feed industry more commonly uses processed crude ginkgo preparations, including leaf powder, ginkgo leaf oil, and fermentation-derived products [142]. Crude preparations are substantially less costly than purified extracts and are therefore economically attractive for large-scale animal production. However, insufficiently processed ginkgo leaf powder often contains high levels of anti-nutritional factors (e.g., phytate, tannins, and oxalates), which may reduce protein and mineral bioavailability; bitterness and off-flavors can also decrease intake in young animals [143]. Solid-state fermentation (SSF), particularly microbial treatment using *Aspergillus niger* or *Candida utilis*, is considered an effective approach to improve the feasibility of crude preparations in feed [142]. Fermentation can increase crude protein by up to 64.9% and markedly reduce GAs; ester compounds produced may improve aroma and palatability, thereby alleviating reduced intake [144]. FGBL can disrupt the dense cell wall matrix to release entrapped flavonoids and terpene lactones; it may also deliver probiotic microbes and metabolites generated during fermentation, potentially conferring additive gut-health effects. Compared with the costly standardized extract EGb 761, FGBL valorizes low-cost leaves or residues and better aligns with the feed industry’s cost–benefit constraints. As an agro-industrial by-product, GBLR is cost-effective; its protein and fiber value suggests potential substitution for alfalfa pellets in ruminant diets, reducing total ration costs without compromising growth performance [126]. In broiler production, adding Ginkgo biloba leaf oil may increase direct supplementation costs per bird; however, it can improve net profit and ROI by lowering FCR, reducing heat-stress mortality, and improving slaughter traits [121].

The efficacy of feed additives depends on an appropriate dosage window. Species-specific metabolic pathways define safety margins and effect magnitude; therefore, dose optimization should consider physiological stage and production objectives. In broilers, inclusion of 3.5–4.5 g/kg of diet FGBL can achieve improved growth performance and muscle antioxidant status [119]. In laying hens, 6 g/kg of the diet FGBL for 42 days is effective in modulating egg cholesterol levels [145]. Unlike poultry, piglet studies often use ginkgo biloba leaf residue as a bulk substitute ingredient; at a 10% inclusion level in the diet, it can functionally promote gut health [123]. In goats, including 18% of the diet GBLR can replace alfalfa as a fiber source, reducing costs and improving rumen health [126]. Another study reported potential hepatoprotective effects of GBE; 7.5 g GBE may reduce oxidative stress and thereby mitigate hepatocellular injury risk (Table 6) [60].

Although ginkgo flavonoids and terpene lactones may be beneficial, ginkgo leaves also contain alkylphenolic acids, i.e., GAs, whose safety risks should not be overlooked. GAs inhibit SUMOylation; at higher exposure levels, they may induce apoptosis and disrupt metabolism, leading to substantial hepatic injury [146]. Moreover, GAs exhibit strong skin and respiratory sensitization potential. During feed processing, dust from crude preparations with elevated GA levels may pose serious occupational health risks; thus, feed-grade GBE should be deacidified to keep GAs below 5 mg/kg feed [147]. SSF is considered among the more industrially feasible approaches, reducing GAs to <5 mg/kg feed while improving the bioavailability of flavonoids. The current literature is heterogeneous in extract standardization, composition, and dosing, and long-term production-scale evidence (including species-specific safety margins and residue considerations in milk/eggs) remains limited. Moreover, mechanistic insights derived from biomedical/in vitro models should be interpreted cautiously and validated in livestock- and aquaculture-relevant feeding trials.

Multidimensional evidence indicates that ginkgo leaf extracts have substantial potential to enhance growth performance, improve carcass quality, alleviate heat stress, and reduce reliance on antibiotics. However, broad regulatory-compliant deployment in the feed industry requires life-cycle residue assessment, clarification of the kinetic distribution of key constituents in milk and egg matrices, and the establishment of food-safety–oriented withdrawal period datasets. The development of industry standards for feed-grade GBE should not be limited to GA restrictions; it should also incorporate proanthocyanidins and specific organic acids into a quality surveillance framework to reduce adulteration risks and ensure batch-to-batch consistency of bioactivity [148]. Collectively, ginkgo leaf extracts may serve not only as a valuable resource in traditional pharmacopeia but also as a strategic tool for achieving antibiotic-free production goals, improving animal welfare, and increasing product value. With rigorous standardization and precision dose management, GBE is poised to play a more pivotal role in sustainable animal nutrition systems.

## 6. Limitations

GBE has multiple biological functions, far exceeding the beneficial biological activities described in this review. Here, we just focus on its biological function, molecular mechanisms, and the application in animal production. Some biological functions of GBE may also contribute to its beneficial effects on animal production, but due to a lack of direct evidence, we did not include them in our review. This is a major limitation of this review. However, narrative reviews have an important role in continuing education because they provide readers with up-to-date knowledge about a specific topic or theme [149].

## 7. Conclusions and Future Perspectives

**Figure 4:**
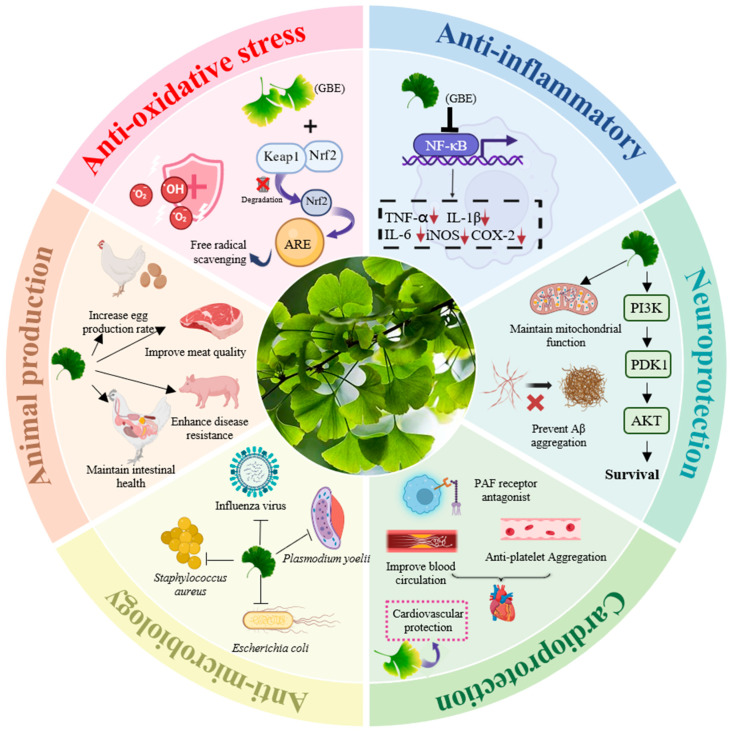
Schematic Diagram of the Pleiotropic Mechanisms of GBE. Core mechanisms of GBE in antioxidation, anti-inflammation, neuroprotection, cardiovascular regulation, immunomodulation, and animal production performance improvement. Created with BioRender.com.

The biological effects of GBE mainly result from its antioxidant, anti-inflammatory, immune-regulating, microcirculatory, and cell-protective activities. These effects are driven by the combined action of several bioactive components, especially flavonoids such as quercetin, kaempferol, and isorhamnetin, as well as terpenoid trilactones including ginkgolides A, B, C, and bilobalide. At the molecular level, GBE acts through several key signaling pathways. First, the activation of the Nrf2/ARE pathway increases the expression and activity of antioxidant enzymes such as SOD, CAT, and GSH-Px, which helps maintain redox balance. Second, the inhibition of excessive NF-κB and MAPK signaling reduces inflammatory responses by lowering the expression of pro-inflammatory cytokines, including TNF-α, IL-1β, and IL-6. Third, the regulation of pattern-recognition receptor pathways such as TLRs/MyD88 affects immune cell activity and cytokine release, which helps maintain immune balance. In addition, GBE can also protect mitochondrial function by inhibiting oxidative stress and apoptosis-related factors such as the Bcl-2/Bax ratio. Together, these effects contributed to the beneficial effects of GBE in enhancing growth performance, stress tolerance, immune function, and product quality in livestock and poultry, including pigs, poultry, and ruminants. This also indicated GBE is a plant-derived feed additive with multiple functions and strong potential to improve animal health, production efficiency, and product quality under intensive farming conditions (as shown in Figure 4).

GBE has strong safety at recommended dosages, with its innate antimicrobial, anti-inflammatory, and immune-enhancing characteristics positioning it as a viable substitute or complementary addition to antibiotic growth promoters. However, significant research gaps remain to be addressed: (1) clarification of the interactions among diverse GBE constituents and the establishment of clear quality benchmarks tied to biological efficacy; (2) deeper mechanistic investigations using multi-omics approaches, particularly those emphasizing intestinal health, gut microbiota, and immune regulation; and (3) large-scale, long-term field trials to optimize dosing strategies, application methods, and combinations with other functional feed additives. Moreover, comprehensive long-term safety studies should evaluate residue profiles and potential interactions with other feed components or medications. Addressing these areas will facilitate more efficient and reliable GBE application in animal production.

## Figures and Tables

**Table 1:** Anti-inflammatory effects of GBE in vitro and in vivo.

Category	Model	Key Findings	Conclusion	Reference
Neuro-protection	Rat hippocampal injury	Decreases MDA/ROS; inhibits NF-κB; decreases TNF-α, IL-1α, IL-6.	Neuronal protection via antioxidant and anti-inflammatory effects.	Kaur et al. [79]
	Mouse epilepsy model	Inhibits lncRNA-COX2/NF-κB; preserves memory performance.	Mitigation of neuroinflammation via lncRNA regulation	Zou et al. [11]
Anti-inflammatory and immune	Mouse colitis model	Decreases iNOS/COX-2/TNF-α; induces effector T-cell apoptosis.	Amelioration of colitis via macrophage modulation	Kotakadi et al. [71]
	LPS-stimulated macrophages	Decreases TLR4; inhibits NF-κB p65; decreases IL-1β/IL-6.	Anti-inflammatory effects via the TLR4/NF-κB pathway	Zhou et al. [72]
	Rat uveitis model	Decreases NO/PGE2/TNF-α; inhibits iNOS expression.	Alleviation of ocular inflammation	Ilieva et al. [73]
	Arsenic-exposed subjects	Modulates Th17/Treg; decreases IL-17A/IL-6; increases IL-10.	Improvement of immune status via T-cell balance	Chen et al. [80]
	Mouse fungal arthritis model	Terpenoid-mediated NO reduction; flavonoid-independent effects	Anti-inflammatory effect by terpenoid fraction	Han et al. [76]
	Asthma-related models	PAF inhibition; airway inflammation suppression	Potential therapeutic application in asthma	Babayigit et al. [81]
Cardiovascular and Metabolic	Hypothyroid/Hypoxic mice	Correction of oxidative imbalance; reduction of inflammatory mediators	Improvement of hypoxia-induced vascular dysfunction.	Adebayo et al. [74]
	Rabbit atherosclerosis model	Decreases ROS; inhibits NF-κB; decreases VCAM-1/ICAM-1.	Suppression of adhesion molecule expression.	Peng et al. [75]
	Hypertensive rats	Decreases blood pressure and improves endothelium-dependent vasodilation.	Long-term improvement of endothelium-dependent dilation	Kubota et al. [82]
	Rat liver injury models	Ginkgolide B attenuates acetaminophen-induced liver injury	PAF antagonism effectively protects the liver from toxic injury.	Grypioti et al. [61]
	Acute pancreatitis models	Ginkgolides inhibit PAF-mediated acinar cell injury	GBE confers protection against PAF-mediated acute pancreatitis.	Gachowska et al. [83]
	Atherosclerosis models	Reduces the formation of atherosclerotic nanoplates and vascular lesion features.	GBE exhibits potential anti-atherosclerotic effects.	Lippi et al. [84]

**Table 4:** Effects of GBE on growth performance in farm animals.

Animal	GBE Form	Dose	Duration	Main Outcomes	Reference
Broilers	Ginkgo biloba leaves (FGBL)	3.5–4.5 g/kg feed	42 days	Increases ADG and ADFI; improves FCR	Niu et al. [119]
Broilers	Ginkgo biloba leaf oil	0.25 cm/L drinking water (i.e., 0.25 mL Ginkgo-biloba-leaf oil per mL water)	3 weeks	Increases final body weight, total weight gain, and feed intake	El-Kasrawyal. et al. [121]
Broilers	Ginkgo biloba extract (GBE)	0.8% (it is equal to 8 g/kg feed)	35 days	Increases weight gain (days 21–35) and improves feed efficiency	Yan et al. [122]
Weaned piglets	Ginkgo biloba leaf residue (GBLR)	10% (it is equal to 100 g/kg feed)	42 days	Increases final body weight and ADG; improves feed efficiency	Zhou et al. [123]
Laying hens	FGBL	0.5% (it is equal to 100 g/kg feed)	8 weeks	Increases egg production and improves FCR	Zhao et al. [124]
Goats (Haimen white)	GBLR	18% (it is equal to 180 g/kg feed)	70 days	Increases final body weight, ADG, and feed intake; improves FCR	Chen et al. [126]
Nile tilapia	GBE	5.0–9.0 g/kg feed	8 weeks	Improves growth performance and feed utilization	Abdel-Latif et al. [60]
Pacific white shrimp	GBE	2 g/kg feed	21 days	Increases survival rate and antioxidant capacity	Liao et al. [127]

**Table 5:** Effects of GBE on meat and egg quality traits.

Animal	GBE Form	Dose	Quality Parameter	Outcome	Reference
Broilers	FGBL	0.2–0.5% (it is equal to 2–5 g/kg feed)	Abdominal fat deposition	Decreases abdominal fat deposition	Cao et al. [129]
Broilers	FGBL	3.5–4.5 g/kg feed	Drip loss (24 h)	Reduces drip loss	Niu et al. [119]
Broilers	FGBL	0.2–0.7% (it is equal to 2–7 g/kg feed)	Muscle fatty acid profile	Decreases SFA content and increases PUFA content	Cao et al. [129]
Broilers	GBE	600 mg/kg body weight	Serum cholesterol	Decreases serum cholesterol	Zhou et al. [14]
Laying hens	FGBL	0.5% (it is equal to 5 g/kg feed)	Yolk cholesterol	Decreases yolk cholesterol	Zhao et al. [124]
Laying hens	FGBL	0.5% (it is equal to 5 g/kg feed)	Yolk fatty acid profile	Increases the PUFA/SFA ratio	Zhao et al. [124]
Finishing pigs	Fermented feed	10% (it is equal to 100 g/kg feed)	Intramuscular fat (IMF)	Increases intramuscular fat content	Song et al. [131]
Goats (Haimen white)	GBLR	18% (it is equal to 180 g/kg feed)	Antioxidant capacity	Enhances antioxidant capacity	Chen et al. [126]

**Table 6:** Summary of GBE and its derivatives: standardization, dosage, and outcomes.

Species	Product Form	Standardization	Dosage	Key Outcome	Reference
Broilers	FGBL	Crude extract	3.5–4.5 g/kg feed	Elevated T-SOD and T-AOC	Niu et al. [119]
Weaned piglets	GBLR	Crude extract	10% GBLR	Increased serum IgA/IgG	Zhou et al. [123]
Laying hens	FGBL	Crude extract	6 g/kg feed	Improved FCR	Zhang et al. [145]
Goats	GBLR	Crude extract	18% GBLR	Reduced feed costs	Chen et al. [126]
Nile tilapia	GBE	Purified extract	7.50 g/kg feed	Increased lysozyme/IgM	Abdel-Latif et al. [60]

## Data Availability

The original contributions presented in this study are included in the article.
